# Correlation Among Body Composition Parameters and Long-Term Outcomes in Crohn's Disease After Anti-TNF Therapy

**DOI:** 10.3389/fnut.2022.765209

**Published:** 2022-04-01

**Authors:** Katsuyoshi Ando, Kyoko Uehara, Yuya Sugiyama, Yu Kobayashi, Yuki Murakami, Hiroki Sato, Takehito Kunogi, Takahiro Sasaki, Keitaro Takahashi, Nobuhiro Ueno, Shin Kashima, Kentaro Moriichi, Hiroki Tanabe, Toshikatsu Okumura, Mikihiro Fujiya

**Affiliations:** ^1^Gastroenterology and Endoscopy, Division of Metabolism and Biosystemic Science, Gastroenterology and Hematology/Oncology, Department of Medicine, Asahikawa Medical University, Asahikawa, Japan; ^2^Cancer Genome, Division of Metabolism and Biosystemic Science, Gastroenterology and Hematology/Oncology, Department of Medicine, Asahikawa Medical University, Asahikawa, Japan; ^3^Division of Metabolism and Biosystemic Science, Gastroenterology and Hematology/Oncology, Department of Medicine, Asahikawa Medical University, Asahikawa, Japan

**Keywords:** Crohn's disease, body composition, skeletal muscle, visceral fat, mesenteric fat index, anti-TNF antibody

## Abstract

**Background:**

The impact of the body composition on the pathophysiology and clinical course of Crohn's disease (CD) has not been fully elucidated.

**Aims:**

To reveal the correlations among body composition and long-term outcomes in CD after anti-TNF therapy.

**Methods:**

*Ninety-one* patients who received anti-TNF therapy as their first biologic treatment were enrolled. The skeletal muscle index (SMI), visceral and subcutaneous fat area (VFA, SFA), and the ratio of the VFA to SFA (mesenteric fat index; MFI) at the 3^rd^ lumbar level were measured using computed tomography (CT) imaging before the induction. The correlation among the body composition and outcomes were retrospectively analyzed.

**Results:**

The 5-year cumulative secondary failure- and resection-free rates in patients with a low SMI (39.1% and 64.8%) were significantly lower than those with a high SMI (67.5% and 92.7%; *p* = 0.0071 and 0.0022, respectively). The 5-year cumulative secondary failure-free rate in the patients with low VF (45.0%) was significantly lower than that in those with high VF (77.6%; *p* = 0.016), and the 5-year cumulative resection-free rate in patients with a high MFI (68.9%) was significantly lower than that in those with a low MFI (83.0%; *p* = 0.031). Additionally, patients with low age and BMI had significantly lower cumulative secondary failure- and resection-free rates than those with high age and BMI (low age: 37.4% and 71.2%; high age: 70.7% and 88.9%; *p* = 0.0083 and 0.027, respectively) (low BMI: 27.2% and 64.8%; high BMI: 68.3% and 87.9%; *p* = 0.014 and 0.030, respectively), respectively. In the multivariate analyses, a low SMI was the only independent risk factor for secondary failure (hazard ratio [HR] 2.15, 95% confidence interval [CI] 1.04–4.44), while low age (HR 4.06, 95% CI 1.07–15.4), a low SMI (HR 4.19, 95% CI 1.01–17.3) and high MFI were risk factors for bowel resection (HR 4.31, 95% CI 1.36–13.7).

**Conclusion:**

The skeletal muscle mass and ratio of visceral to subcutaneous fat were suggested to reflect the long-term clinical outcome and may be helpful as prognostic markers after anti-TNF therapy in CD.

## Introduction

Crohn's disease (CD) is a relapsing and progressive chronic inflammatory intestinal disorder that leads to intestinal stenosis, abdominal abscess, fistula and bowel resection (1). Several studies have reported that more than half of CD patients are complicated with malnutrition, even in a remission state (2, 3). Malnutrition in CD is caused by multiple factors, including decreased oral food intake, medications such as corticosteroids, malabsorption, and increased energy expenditure (4). Malnutrition with CD is strongly associated with micronutrient and vitamin deficiency and alterations of the body composition including subcutaneous and visceral fat (SF and VF) and skeletal muscle mass (4). However, previous studies have reported that the body mass index (BMI) does not always reflect the body composition, as defined by fat and skeletal muscle mass, in CD patients (3, 5–7). In addition, alterations of subcutaneous and visceral fat and skeletal muscle mass in CD have been suggested to be regarded as biomarkers that reflect the disease phenotype (8, 9), activity (9), treatment response (7) and outcome, including bowel resection (10–12) and complications after surgery (13, 14). Therefore, the measurement of fat and skeletal muscle mass in CD were suggested to be crucial for predicting the disease course, as well as BMI and disease activity. Recently, the measurement and analysis of fat and skeletal muscle using abdominal computed tomography (CT) at the third lumbar level have been reported to be reliable and to have potential application as predictive biomarkers for not only inflammatory bowel disease, including CD (15–17), but also cancer, obesity and liver disease (18–21). Abdominal computed tomography (CT) is routinely utilized to assess the extent and complications of disease in clinical practice for CD. Therefore, abdominal CT is a more convenient and accessible tool for the analysis of the body composition in routine care for CD patients in comparison to whole body dual-energy X-ray absorptiometry (DXA).

According to a meta-analysis, the risk of surgery in CD at 5 and 10 years after the diagnosis of CD has been reported to be as high as 33.3 and 46.6%, respectively (22); however, the rate of surgery in CD has decreased over time (22, 23). Previous studies have suggested that the earlier induction of anti-TNF therapy remarkably contributed to a reduction in the surgery rate in CD (24–26). However, the annual rate of secondary loss of response (LOR) to anti-TNF therapy per patient-year in CD is reported to be 13–20% (27). Various factors have been reported to predict a non-response to anti-TNF therapy, including disease-related marker including fecal calprotectin, smoking, serological and genetic markers (28), while the association with the body composition has not been fully elucidated. The BMI at the induction has been revealed as a predictor of the drug concentration after 6 months and dose escalation (29, 30). A previous report revealed that a low muscle mass at the initiation of anti-TNF therapy for IBD was a risk factor for a LOR to anti-TNF therapy in the short term (31). However, no studies analyzing the correlation among body composition and long-term outcomes, including the need for surgery, as well as the rate of secondary failure in anti-TNF naïve CD patients have been demonstrated. This study aimed to reveal the correlation among body composition and long-term outcomes in patients with CD after anti-TNF therapy.

## Methods

### Study Population

A total of 200 consecutive anti-TNF naïve CD patients in whom anti-TNF-α antibody therapy had been initiated were identified from a chart review at Asahikawa Medical University Hospital (Japan) between January 2007 and December 2018. Among them, 91 patients with anti-TNF-naïve CD in whom anti-TNF-α antibody therapy was initiated, and who met the following criteria, were enrolled in this study: (1) anti-TNF antibody was induced for the treatment of intestinal symptoms and/or lesions, (2) observational period after TNF therapy ≥1 year, (3) computed tomography was performed within 1 month before the administration of anti-TNF-α antibody therapy. Patients with only perianal disease or extraintestinal manifestations were excluded. This study retrospectively analyzed the correlation among the body composition parameters and long-term clinical outcomes after the induction of anti-TNF therapy in CD.

This study protocol was approved by the Ethics Committee of Asahikawa Medical University (Registry Number: 18235). Informed consent was obtained by announcing this study on the web and providing an opportunity to opt out.

### Data Collection

The following patient characteristics before the administration of anti-TNF-α antibody therapy were collected from the patients' medical records: age, sex, height, body weight (BW), body mass index (BMI), type of disease extension (ileitis, ileo-colitis, colitis type), disease duration (from the development of CD), disease duration until the induction of anti-TNF-α antibody therapy, type of anti-TNF-α antibody (infliximab or adalimumab), presence or absence of concomitant immunomodulator usage. The following laboratory data before the administration of anti- TNF-α antibody therapy were also collected from medical records: white blood cell, neutrophil and lymphocyte counts, and the serum levels of C-reactive protein, albumin, total cholesterol. The periods until the occurrence of secondary failure and/or bowel resection were collected from medical records, which were regarded as indicators of the long-term clinical outcome after the administration of anti-TNF-α antibody therapy. If a loss of response was suspected in clinical practice, endoscopy (ileocolonoscopy with or without balloon or capsule enteroscopy) and/or imaging studies (ultrasonography, CT and magnetic resonance imaging) in combination with a stool culture test (including clostridium) was performed to confirm the presence of worsening of endoscopic and/or imaging (transmural and extraintestinal) findings and to rule out the enteral infection. Secondary failure was defined by the following criteria (1 + [2 and/or 3]: (1) loss of response (worsening of clinical symptoms in combination with endoscopic and/or imaging findings) after a primary response to anti-TNF-α antibody therapy (after the induction phase), (2) strengthening and/or alteration of anti-TNF therapy, which included increasing the dose and shortening the duration of anti-TNF-α antibody therapy, alteration of biologics and the addition of concomitant therapy (immunomodulator, steroids and cytapheresis), and (3) the aggravation of gastrointestinal lesions on endoscopy and/or surgical specimens. In addition, bowel resection was defined as resection of the small and/or large intestine due to worsening intestinal symptoms and/or lesions after the induction of anti-TNF-α antibody therapy with or without stoma construction.

### Measurements of the Skeletal Muscle and Fat Area

The skeletal muscle area (SMA) [cm^2^], psoas muscle area (PMA) and visceral and subcutaneous fat area (VF [cm^2^], SF [cm^2^]) in addition to abdominal circumference (AC [cm]) at the 3rd lumbar level were measured from CT imaging before the induction of anti-TNF therapy, using the SYNAPSE VINCENT software program (Fuji Film, Tokyo, Japan). The skeletal muscle mass index (SMI [cm^2^/m^2^]) and psoas muscle index (PMI [cm^2^/m^2^]) was calculated as the respective muscle area divided by the square of height. The mesenteric fat mass index (MFI) was calculated as the ratio of VF to SF. SMA/SF was calculated as the ratio of SMA to SF.

### Statistical Analyses

All statistical analyses were performed using EZR (Saitama Medical Center, Jichi Medical University, Saitama, Japan), which is a graphical user interface for R (The R Foundation for Statistical Computing, Vienna, Australia). The difference in measurements between male and female patients was compared with an unpaired *t*-test. The cut-off values of measurements for classification into two groups were determined with a receiver operating characteristics (ROC) analysis, the outcome of which was established as the presence or absence of secondary failure. Cut-off values were determined based on the threshold nearest to the top-left corner of the ROC curve. The cumulative secondary failure-free and bowel resection-free rates were calculated with the Kaplan-Meier method. The cumulative secondary failure-free and bowel resection-free rates were classified into two groups based on cut-off values and the groups were compared with a log-rank test. Bonferroni correction was applied in cases involving the comparison among more than three groups. The risk factors for secondary failure and bowel resection were analyzed with a Cox proportional hazards model, which included parameters that were identified as significant in a log-rank test. *P* < 0.05 were considered to indicate statistical significance.

## Results

### Characteristics and Measurements of the Patients

The characteristics and laboratory data of 91 patients with CD before the induction of anti-TNF-α antibody therapy are summarized in Table 1. The body composition measurements are described in Supplementary Table 1. The average of VFA was 41.6 ± 38.1 (mean ±SD) (Male; 44.6 ± 38.2, Female; 32.1± 37.4, *p* = 0.183), the average SFA was 65.0 ± 55.8 (M; 59.8 ± 54.9, F; 81.0± 56.4, *p* = 0.122), and the average MFI was 0.82 ± 0.59 (M; 0.91 ± 0.54, F; 0.53± 0.64, *p* = 0.0078). The average SMA was 111.3 ± 30.5 (M; 122.1 ± 25.8, F; 77.6± 16.1, *p* < 0.0001), the average SMI was 39.8 ± 9.1 (M; 42.6 ± 8.3, F; 31.1 ± 5.2, *p* < 0.0001), PMA was 15.7 ± 5.6 (M; 17.8 ± 4.4, F; 9.0± 2.8, *p* < 0.0001), and the average PMI was 5.5 ± 1.8 (M; 6.1 ± 1.6, F; 3.6 ± 1.0, *p* < 0.0001). The average SMA/SF was 3.5 ± 3.7 (M; 4.1 ± 4.0, F; 1.5 ± 1.0, *p* = 0.0029). All measurements associated with skeletal muscle area showed significant differences between males and females while the actual measurement value of the visceral and subcutaneous fat areas did not differ to a statistically significant extent despite a higher tendency in males. The cut-off value of SMI was established as sex-specific because sex-specific cut-off values of skeletal muscle areas have commonly been used for certain investigations.

**Table 1:** Patient demographics.

Age at the induction of anti-TNF-α antibody therapy (years) (mean ± SD)	32.7 ± 14.9
Sex (M/F)	69/22
Height (cm) (mean ± SD)	166.2 ± 8.5
Body weight (kg) (mean ± SD)	55.8 ± 11.8
BMI (kg/m^2^) (mean ± SD)	20.1 ± 3.3
AC (cm) (mean ± SD)	74.3 ± 9.3
Disease background at the induction of anti-TNF-α antibody therapy	
Type of disease (I/IC/C)	20/54/17
Disease duration (Months) (mean ± SD)	144.4± 106.4
Disease duration until the induction of anti-TNF-α antibody therapy (Months) (mean ± SD)	60.4 ± 78.3
Type of anti-TNF-α antibody	
Infliximab/Adalimumab, N (%)	74 (81.3%) /17 (18.7%)
Concomitant immunomodulator usage, N (%)	21 (23.1 %)
Average observation period (months)	61.9 ± 41.5
Laboratory data before the induction of anti-TNF-α antibody therapy	
WBC (/μl) (mean ± SD)	5,846 ± 2,475
TLC (/μl) (mean ± SD)	1,090 ± 490
Serum Alb (g/dl) (mean ± SD)	3.5 ± 0.59
T-Chol (mg/dl) (mean ± SD)	131.3 ± 28.1
CRP (mg/dl) (mean ± SD)	2.0 ± 3.3

*BMI, Body mass index; AC, Abdominal circumference; I/IC/C, Ileitis/Ileo-colitis/Colitis; WBC, White blood cell count; TLC, Total lymphocyte count; Alb, Albumin; T-Chol, Total cholesterol; CRP, C-reactive protein*.

### Cut-Off Values Based on ROC Analysis

ROC analyses were performed to determine cut-off values for secondary failure of anti-TNF-α antibody therapy. The cut-off values were determined based on the threshold nearest to the top-left corner of the ROC curve, and were as follows: age at the induction of anti-TNF-α, threshold 32, AUC 0.6907; BW, 56.3, AUC 0.5916; BMI, threshold 19.0; AUC 0.661; AC, threshold 71.2, AUC 0.616; duration of disease, threshold 180.0, AUC 0.576; disease duration until the induction of anti-TNF-α antibody therapy, threshold 49, AUC 0.569; WBC, threshold 6060, AUC 0.532; TLC, threshold 1030, AUC 0.492; serum Alb, threshold 3.4, AUC 0.579; TC, threshold 125, AUC 0.540; CRP, threshold 3.22, AUC 0.492; VFA, threshold 50.8, AUC 0.603; SFA, threshold 50.8, AUC 0.438; MFI, threshold 0.77, AUC 0.554; SMI, threshold of males 43.2, threshold of females 31.0, AUC of males 0.547, threshold of females 0.650, male; and SMA/SF, threshold 2.2, AUC 0.457.

### Comparison of Cumulative Secondary Failure-Free and Bowel Resection-Free Rates Classified According to Body Composition Measurements

**Figure 1:**
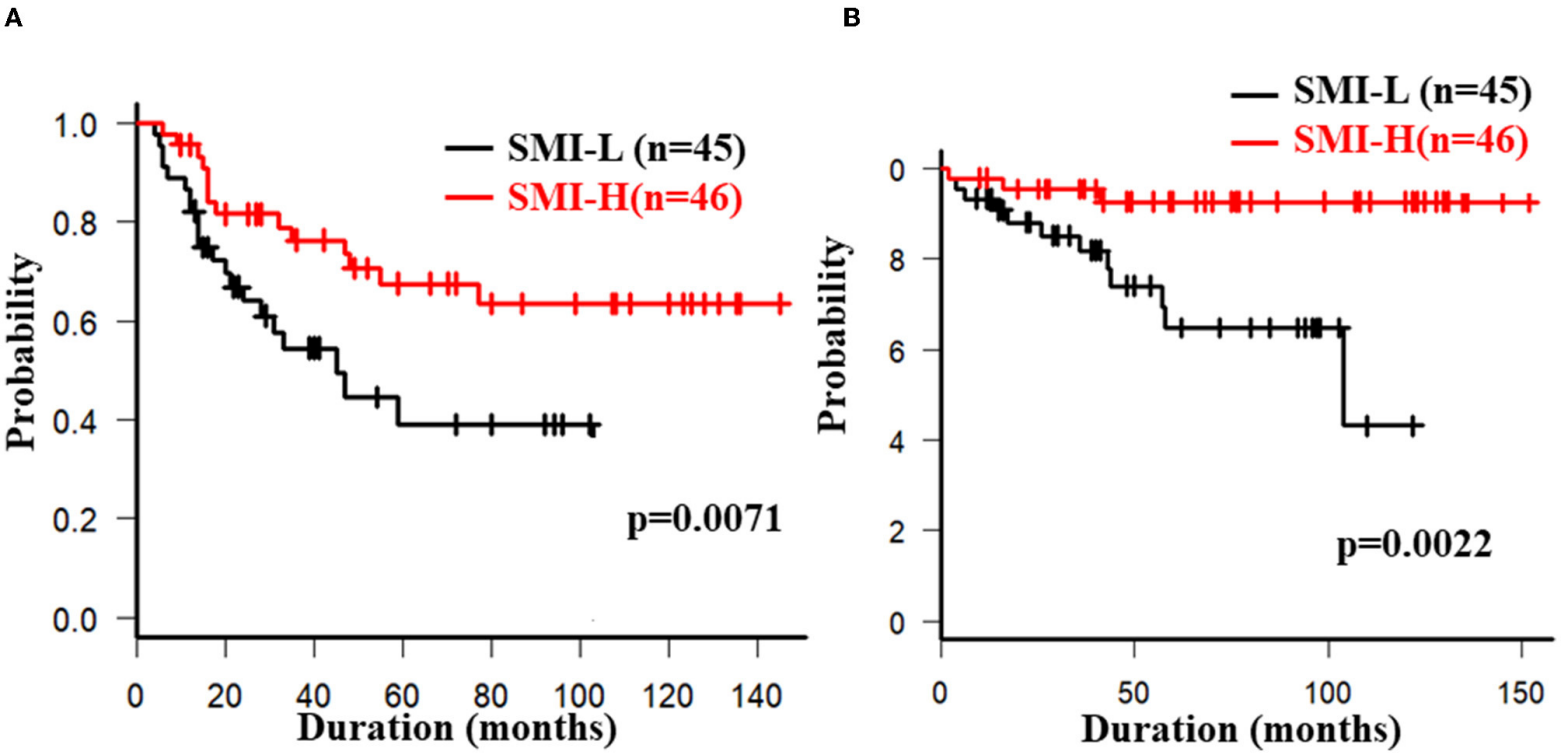
Cumulative secondary failure-free **(A)** and bowel resection-free **(B)** rates classified according to the SMI.

**Figure 2:**
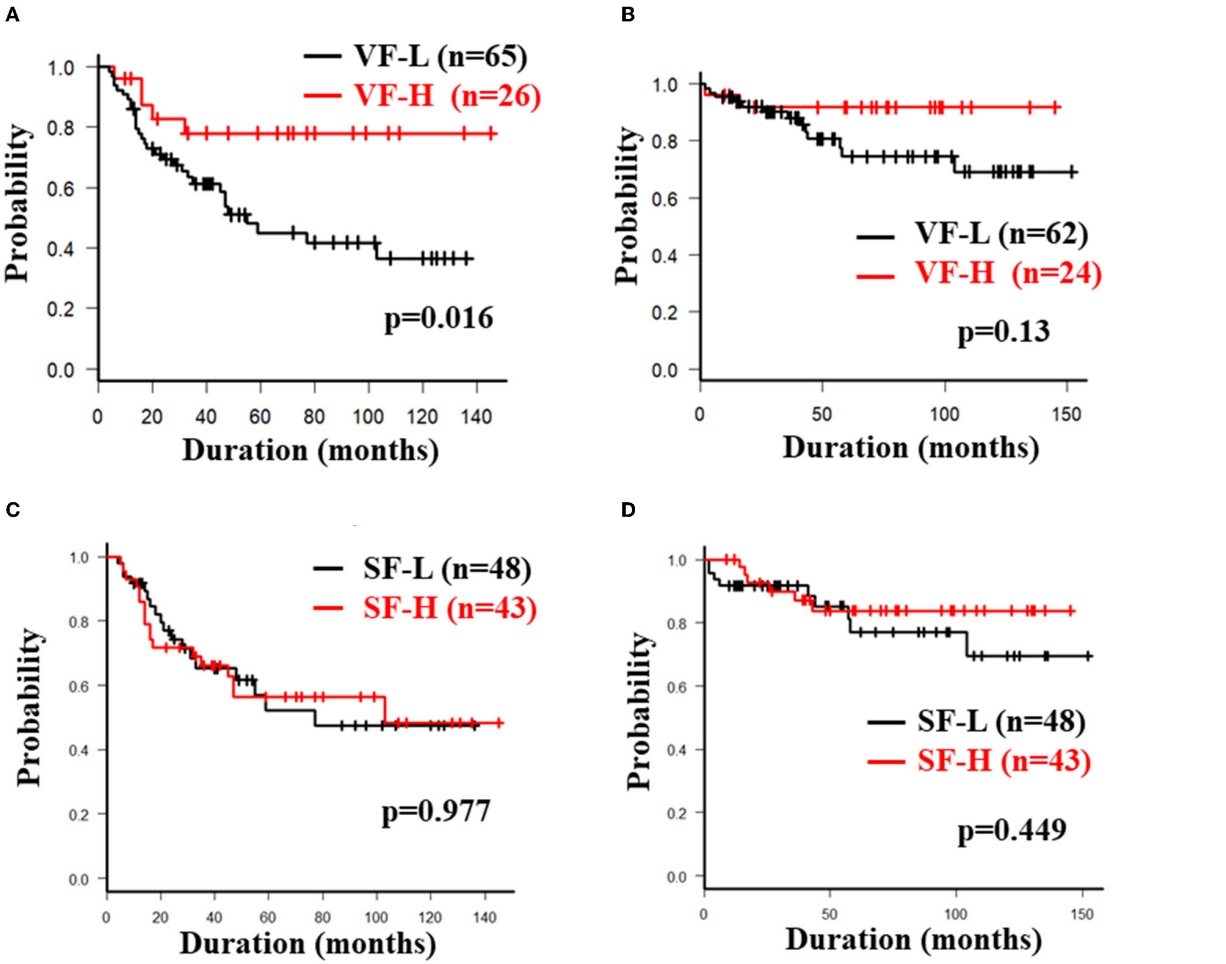
Cumulative secondary failure-free and bowel resection-free rate classified according to the fat mass. Each figure shows the cumulative secondary failure-free rate **(A)** and bowel resection-free rate **(B)** classified according to VF, and the cumulative secondary failure-free rate **(C)** and bowel resection-free rate **(D)** classified according to SF.

**Figure 3:**
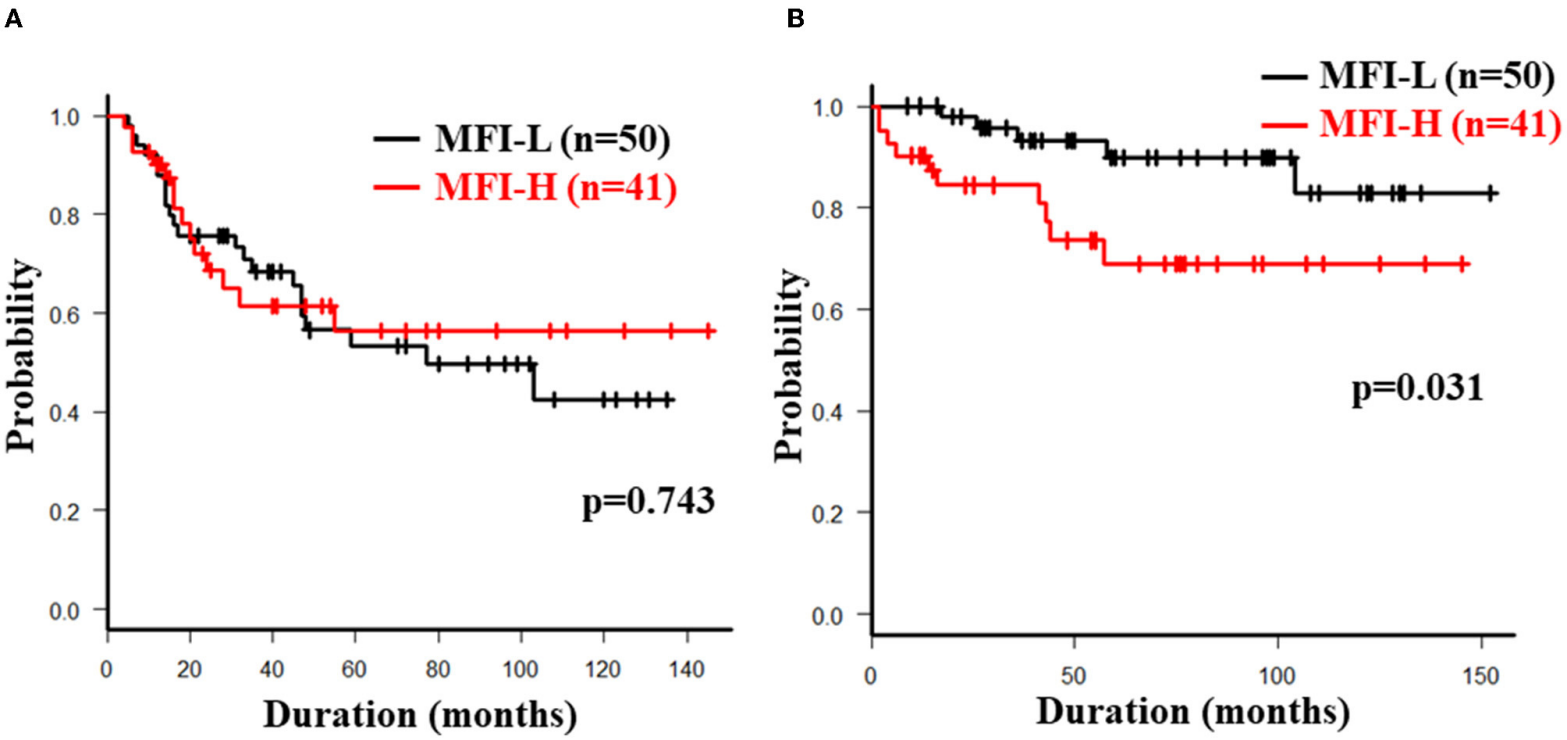
Cumulative secondary failure-free **(A)** and bowel resection-free **(B)** rates classified according to the MFI.

The overall 5-year cumulative secondary failure-free and bowel resection-free rates after the induction of anti-TNF therapy were 55.0% and 80.4%, respectively (Supplementary Figures 1A,B). The 5-year cumulative secondary failure-free and bowel resection-free rates in patients with a low SMI were 39.1 and 64.8%, respectively, which were significantly lower than those in patients with a high SMI (67.5% and 92.7%; *p* = 0.0071 and 0.0022, respectively) (Figures 1A,B). The 5-year cumulative secondary failure-free rate in patients with low VF (45.0%) was lower than that in patients with high VF (77.6%; *p* = 0.016), and the 5-year bowel resection-free rate in patients with low VF (74.5%) tended to be low in comparison to that in patients with high VF (91.8%; *p* = 0.13) (Figures 2A,B). The 5- year cumulative secondary failure-free rate and bowel resection-free rates were not affected by SFA (Figures 2C,D). The 5-year bowel resection-free rate in patients with a high MFI (68.9%) was significantly lower than that in patients with a low MFI (83.0%; *p* = 0.031), while the 5-year secondary failure-free rates in two groups did not differ to a statistically significant extent difference (Figures 3A,B). When classified into four groups based on the combination of low-and high- SMI and VF, the 5-year cumulative secondary failure-free rate in the high SMI and high VF group was 77.0%, which tended to be high in comparison to the other three groups. Additionally, the 5-year cumulative bowel resection-free rate in the low SMI and low VF group (51.3%) was significantly lower than that in the high SMI and low VF group (92.0%; *p* = 0.027) (Supplementary Figures 2A,B).

### Comparison of Cumulative Secondary Failure-Free and Bowel Resection-Free Rates With Other Measurements and Patient Characteristics

**Figure 4:**
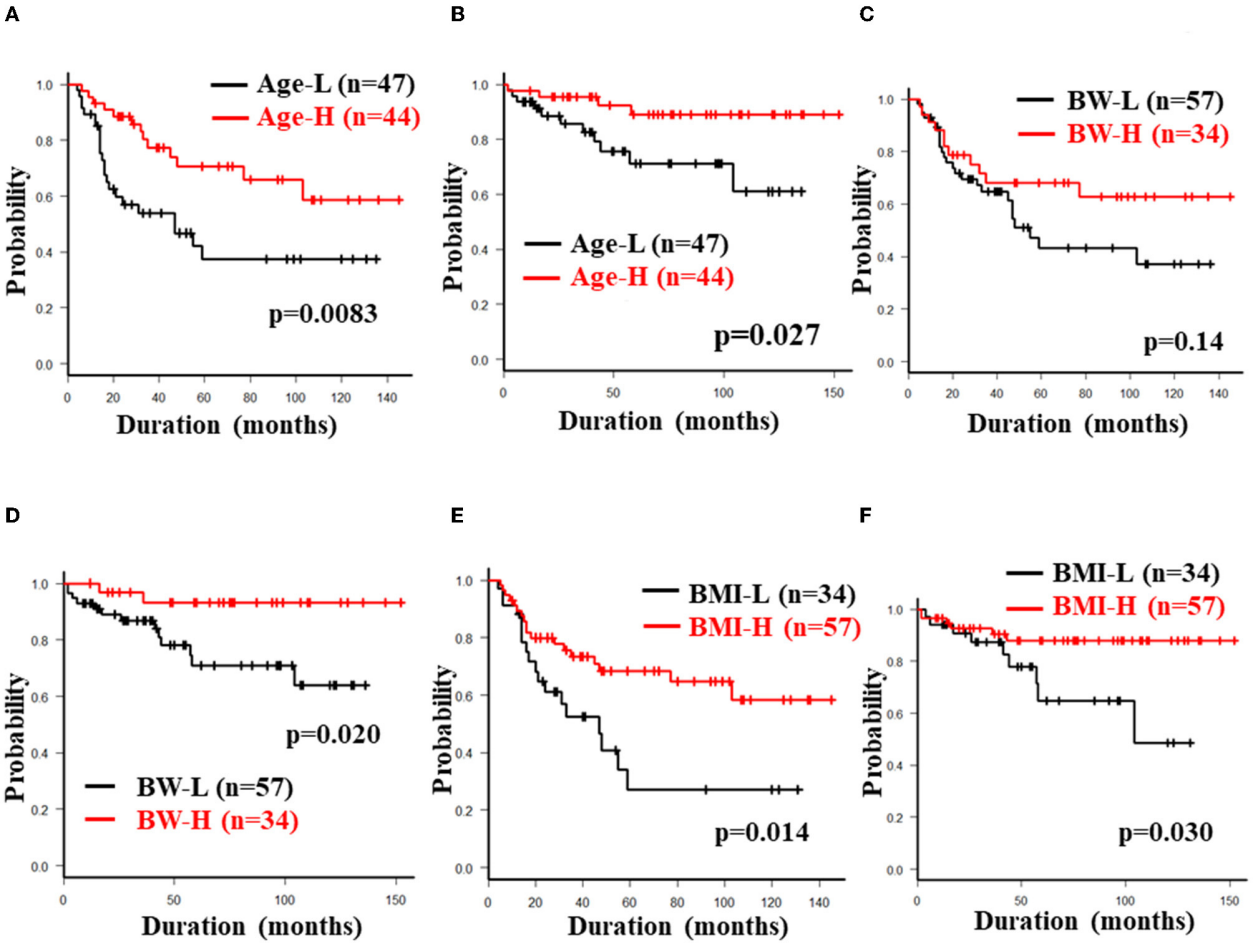
Cumulative secondary failure-free and bowel resection-free rates classified according to age at induction, BW and BMI. The figures show the cumulative secondary failure-free rate **(A)** and bowel resection-free rate **(B)** classified according to the age at induction, the cumulative secondary failure-free rate **(C)** and bowel resection-free rate **(D)** classified according to BW, and the cumulative secondary failure-free rate **(E)** and bowel resection-free rate **(F)** classified according to BMI.

The 5-year cumulative secondary failure-free and bowel resection-free rates in patients who were <32 years of age at the induction of anti-TNF-α antibody therapy were 37.4% and 71.2%, respectively, which were significantly lower in comparison to those who were ≥32 years of age (70.7% and 88.9%; *p* = 0.0083 and 0.027, respectively) (Figures 4A,B). The 5-year bowel resection-free rate in patients whose body weight (BW) was <56.3 kg at the induction of anti-TNF-α antibody therapy was 71.0%, which was significantly lower in comparison to that in patients whose BW was ≥56.3 kg (91.3%; *p* = 0.020) (Figures 4C,D). The 5-year cumulative secondary failure-free and bowel resection-free rates in patients with BMI <19.0 at the induction of anti-TNF-α antibody therapy were 27.2 and 64.8%, respectively, which were significantly lower than those in patients with BMI ≥19.0 (68.3% and 87.9%; *p* = 0.014 and 0.030, respectively) (Figures 4E,F). No other characteristics or measurements affected the 5-year cumulative secondary failure-free or bowel resection-free rates (Tables 2A, 3A).

**Table 2A:** Univariate analyses of factors associated with secondary failure during anti-TNF therapy (log rank test).

	**5-year cumulative secondary failure-free rate**	***p*-value**
Age at induction of anti-TNF-α antibody <32 years	37.4%	0.0083
≥32 years	70.7%	
Male	57.0%	0.85
Female	44.9%	
Body weight <56.3 kg	43.3%	0.14
≥56.3kg	68.0%	
BMI <19.0 kg/m^2^	27.2%	0.014
≥19.0 kg/m^2^	68.3%	
AC <70.6 cm	42.4%	0.22
≥70.6 cm	63.5%	
Disease background at induction of anti-TNF-α antibody therapy	
Type of disease (I/IC/C)	60.6%/50.4%/57.8%	0.89
Duration of disease <180 months	54.1%	0.39
≥180 months	55.0%	
Duration until the induction of anti-TNF-α antibody therapy <49 months	37.4%	0.96
≥49 months	70.7%	
Type of anti-TNF-α antibody therapy - Infliximab	57.3%	0.61
-Adalimumab	43.3%	
Concomitant immunomodulator usage: Yes	74.0%	0.11
No	46.4%	
Laboratory data before the induction of anti-TNF-α antibody	
WBC <6060/μl	59.2%	0.14
≥6060/μl	53.8%	
TLC <1030/μl	54.2%	0.69
≥1030/μl	61.8%	
Serum Alb <3.4 g/dl	54.1%	0.91
≥3.4 g/dl	62.2%	
T-Chol <125 mg/dl	57.5%	0.72
≥125mg/dl	57.7%	
CRP <3.2 mg/dl	57.5%	0.26
≥3.2mg/dl	58.3%	
Body composition parameters	
VFA <50.8 cm^2^	45.0%	0.016
≥50.8	77.9%	
SFA <50.8 cm^2^	52.1%	0.977
≥50.8 cm^2^	56.3%	
MFI >0.77	53.4%	0.743
≥0.77	56.3%	
SMI <43.2 cm^2^/m^2^ (male), 31.0 cm^2^/m^2^ (female)	39.1%	0.0071
≥43.2 cm^2^/m^2^ (male), 31.0 cm^2^/m^2^ (female)	67.5%	
SMA/SFA <2.2	51.7%	0.518
≥2.2	57.8%	

*BMI, Body mass index; AC, Abdominal circumference; I/IC/C, Ileitis/Ileo-colitis/Colitis; WBC, White blood cell count; TLC, Total lymphocyte count; Alb, Albumin; T-Chol, Total cholesterol; CRP, C-reactive protein; VFA, Visceral fat area; SFA, Subcutaneous fat area; MFI, Mesenteric fat index; SMA, Skeletal muscle area; SMI, Skeletal muscle index*.

**Table 2B:** Multivariate analyses of factors associated with secondary failure during anti-TNF therapy (Cox proportional hazards analysis).

	**Multivariate analysis**		
	**HR**	**95% CI**	***p* value**
Younger age (Age at the induction of anti-TNF-α antibody <32 years)	1.93	0.93–3.97	0.076
Low BMI (BMI <19.0 kg/m^2^)	1.18	0.56–2.51	0.65
Low VFA (VFA <50.8 cm^2^)	1.92	0.67–5.49	0.23
Low SMI [SMI <43.2 cm^2^/m^2^ (male), 31.0 cm^2^/m^2^ (female)]	2.15	1.04–4.44	0.039

**Table 3A:** Univariate analysis of factors associated with bowel resection during anti-TNF therapy (log rank test).

	**5-year cumulative bowel resection-free rate**	***p*-value**
Age at the induction of anti-TNF-α antibody <32 years	71.2%	0.027
≥32 years	88.9%	
Male	78.1%	0.68
Female	87.5%	
Body weight <56.3 kg	71.0%	0.020
≥56.3kg	93.1%	
BMI <19.0 kg/m^2^	64.8%	0.030
≥19.0 kg/m^2^	87.9%	
AC <70.6 cm	75.8%	0.71
≥70.6 cm	83.0%	
Disease background at the induction of anti-TNF-α antibody therapy	
Type of disease (I/IC/C)	89.6%/69.0%/85.7%	0.15
Duration of disease <180 months	64.6%	0.28
≥180 months	80.1%	
Duration until the induction of anti-TNF-α antibody therapy <49 months	82.9%	0.80
≥49 months	76.7%	
Type of anti-TNF-α antibody - Infliximab	78.3%	0.35
-Adalimumab	92.8%	
Concomitant immunomodulator usage; Yes	85.6%	0.36
No	72.7%	
Laboratory data before the induction of anti-TNF-α antibody therapy	
WBC <6060 /μl	76.4%	0.89
≥6060 /μl	78.2%	
TLC <1030/μl	76.1%	0.76
≥1030/μl	83.0%	
Serum Alb <3.4 g/dl	79.4%	0.28
≥3.4 g/dl	73.0%	
T-Chol <125 mg/dl	64.1%	0.26
≥125mg/dl	83.3%	
CRP <3.2 mg/dl	76.0%	0.82
≥3.2mg/dl	75.0%	
Body composition parameters	
VFA <50.8 cm^2^	91.8%	0.13
≥50.8	74.5%	
SFA <50.8 cm^2^	77.1%	0.449
≥50.8 cm^2^	83.8%	
MFI >0.77	89.9%	0.031
≥0.77	68.9%	
SMI <43.2 cm^2^/m^2^ (male), 31.0 cm^2^/m^2^ (female)	69.4%	0.0022
≥43.2 cm^2^/m^2^ (male), 31.0 cm^2^/m^2^ (female)	84.5%	
SMA/SFA <2.2	81.7%	0.88
≥2.2	91.6%	

*BMI, Body mass index; AC, Abdominal circumference; I/IC/C, Ileitis/Ileo-colitis/Colitis; WBC, White blood cell count; TLC, Total lymphocyte count; Alb, Albumin; T-Chol, Total cholesterol; CRP, C-reactive protein; VFA, Visceral fat area; SFA, Subcutaneous fat area; MFI, Mesenteric fat index; SMA, Skeletal muscle area; SMI, Skeletal muscle index*.

### The Risk Factors According to the Multivariate Analysis

In a multivariate analysis with a Cox proportional hazards model, low SMI (hazard ratio 2.15, 95% confidence interval 1.04-4.44, *p* = 0.039) was the only independent risk factor for secondary failure. A low SMI was also detected as an independent risk factor for bowel resection (hazard ratio 4.19, 95% confidence interval 1.01–17.3, *p* = 0.048), suggesting that its useful as a marker for predicting the long-term outcomes in CD patients treated with anti-TNF-α therapy. Age <32 years at the induction of anti-TNF-α antibody therapy (hazard ratio 4.06, 95% confidence interval 1.07–15.4, *p* = 0.039) and MFI>0.77 (hazard ratio 4.31, 95% confidence interval 1.36–13.7, *p* = 0.013) were detected as independent risk factors for bowel resection (Tables 2B, 3B).

**Table 3B:** Multivariate analysis of factors associated with bowel resection during anti-TNF therapy (Cox proportional hazards analysis).

	**Multivariate analysis**		
	**HR**	**95% CI**	***p* value**
Younger age (Age at the induction of anti-TNF-α antibody <32 years)	4.06	1.07–15.4	0.039
Low BW (BW <56.3 kg)	3.63	0.61–21.5	0.16
Low BMI (BMI <19.0 kg/m^2^)	0.52	0.13–2.07	0.35
Low MFI (MFI >0.77)	4.31	1.36–13.7	0.013
Low SMI [SMI <43.2 cm^2^/m^2^ (male), 31.0 cm^2^/m^2^ (female)]	4.19	1.01–17.3	0.048

## Discussion

This is the first report to elucidate the correlation between altered body composition (skeletal muscle, visceral fat mass and distribution of visceral and subcutaneous fat) and long-term outcomes (need for surgery as well as secondary failure) over 5 years after anti-TNF therapy in anti-TNF-naïve CD. Our multivariate analysis clearly indicated that a low SMI was associated with a low cumulative secondary failure-free rate and bowel resection free-rate after anti-TNF therapy. A significant difference was observed in the SMI values of male and female patients in our study. Therefore, the sex-specific cut-off values for the SMI were established in our study. In addition to the present study, sex-specific cut-off values have commonly been used for the SMI in other studies. In the Cox proportional model that included sex, a low SMI was still identified as an independent risk factor for both secondary failure (HR 2.16, 95% CI 1.02-4.60, *p* = 0.044) and bowel resection (HR 2.18, 95%CI 1.03-4.65, *p* = 0.043). We therefore concluded that a low SMI was an independent risk factor, irrespective of sex.

Very few studies have shown the relationship between the status of skeletal muscle and the outcomes of CD patients treated with anti-TNF therapy. Ding et al. showed that the presence of myopenia was an independent predictor of a primary non-response to anti-TNF therapy in CD (7). Holt et al. proposed that low muscle mass at the initiation of anti-TNF therapy for IBD was identified as a risk factor for a secondary loss of response to anti-TNF therapy while the average observational period was approximately 2 years and the surgery rate was not mentioned (31). Considering our results and previous reports, a low SMI before the initiation of anti-TNF therapy is a useful predictive marker of the long-term outcomes associated with primary and secondary loss of response and the need for abdominal surgery. Interestingly, the previous report suggested that the trough level of adalimumab was negatively correlated with the body surface area and muscle parameters, although the study population was relatively small (32). The trough levels of anti-TNF-α antibodies and neutralizing antibodies to anti-TNF-α antibodies have been known to be associated with a secondary loss of response to anti-TNF-α antibodies. Further studies are required to elucidate the correlation among these parameters, body composition, and outcomes.

Previous studies have suggested that, under chronic inflammation, a decreasing level of IGF-1 caused by TNF-α and IL-6, an increasing level of myostatin, known as myokine, secreted from skeletal muscle and proinflammatory cytokines, including TNF-α itself, reduced muscle synthesis and enhanced muscle degradation (4). Indeed, Subramaniam et al. reported that anti-TNF therapy reverses sarcopenia in patients with CD (33). This suggests that inflammation- and muscle-associated mediators altered by chronic inflammation, including CD mediate the reduction of skeletal muscles. Notably, a myokine, irisin, has been reported to ameliorate experimental colitis and reduce the colonic TNF-α concentration (34), suggesting that the skeletal muscle condition is a new treatment target for CD, as well as a predictive marker of the effect of anti-TNF therapy. Further investigation concerning the interaction between skeletal muscle and chronic inflammation, particularly the influence of muscle- and inflammation-associated mediators, is needed to elucidate the pathophysiology as well as novel biomarkers and treatment targets in CD.

Our univariate analysis demonstrated that the cumulative secondary failure-free and bowel resection-free rates were higher in CD with a low absolute volume of VF. However, in our multivariate analysis, VF was not reported to be an independent risk factor for the clinical outcomes, while the MFI, which reflected the fat distribution, was detected. These results suggest that the fat distribution is more important than the absolute volume of visceral and subcutaneous fat for predicting the response to anti-TNF therapy. The findings of most previous reports concerning absolute visceral fat and disease activity and the outcomes of CD conflicted with our results (9, 15, 35, 36). Only one report showed that lower visceral fat in CD predicted an increasing rate of surgery or mortality (12). This discrepancy between our results and previous studies was considered due to the diversity of the absolute VF volume in the patients of each study. For example, Shen et al. reported in another Asian study that a low VFA was associated with mucosal healing after anti-TNF therapy and good clinical outcomes. However, the average VFA in the low VFA group (47.7 cm^2^) was almost the same as that of the high VFA group in our study (48.6 cm^2^). Furthermore, the average VFA of the high VFA group in the study of Shen et al. was much higher than that of the high VFA group in our study (35). In addition, according to studies from Western countries (15), the average VFA in enrolled CD patients was much higher than that in our study and other Asian studies. These data indicated that the absolute volume of VF was dependent on the patient background factors, including region, race and dietary habits (37). Infliximab clearance was found to be increased in IBD patients with high (>120 kg) and low (<40 kg) body weight (38), which suggests that an appropriate volume of VF maintained the proper concentration of anti-TNF antibody in CD patients. In a previous study analyzing the correlation between the MFI and disease behavior, the MFI in complicated CD (fistulating and stricturing type) was significantly higher than that in uncomplicated CD and the MFI was only an independent predictor of complicated behavior of CD; accordingly it was reported to be a possible predictive marker for aggressive CD (8). Furthermore, a high proportion of visceral fat to total fat mass, which indicated a high proportion of visceral to subcutaneous fat, has been reported to be associated with an increasing level of proinflammatory cytokine and disease activity (9). Thus, the MFI has been suggested to be affected with natural history of CD. Our study reported that CD in the high MFI group was a predictive factor for bowel resection while a high absolute VF value in CD was a negative predictive factor for the long-term prognosis. This discrepancy emphasizes the importance of fat distribution. Further studies are needed to determine the appropriate range of the absolute VF volume and MFI in individual patients.

Mesenteric adipocytes have been known to release adipokines, such as leptin and adiponectin, and proinflammatory cytokines such as TNF-α, IL-6 and IL-1β (39). Previous research revealed that excessive mesenteric fat in CD exacerbated intestinal inflammation via an imbalance of adipokine, increasing the levels of proinflammatory cytokines and macrophage infiltration (39). Additionally, crosstalk between adipokine-releasing adipocytes and myokine-releasing skeletal muscle have been reported in experimental colitis model (40). The role of skeletal muscle and adipocytes in the pathophysiology of CD needs to be elucidated and the new biomarkers and therapeutic targets most be identified.

In our study, young age (<32 years) at the induction of anti-TNF therapy was a positive predictive factor for bowel resection in multivariate analyses. A meta-analysis by Zhang et al. suggested that younger age at the onset of CD was a predictor of an LOR to infliximab (41). Patients in whom anti-TNF therapy was initiated at a young age were thought to have severe inflammation. Additionally, younger age was reported to be an independent risk factor for a non-response to anti-TNF antibody treatment in surgically-treated patients with biologic-naïve stricturing CD (42). These reports indicated that younger age at the onset of CD was a risk factor for primary and secondary failure of anti-TNF therapy.

The present study was associated with some limitations. First, this was a retrospective study with single institutional cohort and a relatively small study population. However, the average observational period in this study was >5 years, which was much longer in comparison to previous studies. Additionally, two different outcomes (secondary failure and bowel resection) were investigated in our study. Furthermore, TNF-naïve CD patients were specifically selected, while previous reports included less specific cohorts, such as hospitalized patients and patients with a history of various medical treatments. Secondly, the cut-off values of the body composition measurements were established with an ROC analysis; thus, therefore, the cut-off values in our study might not be useful in other cohorts, especially Western countries. However, at present, the gold-standard cut-off values of skeletal muscle and fat mass, which might be dependent on region, race and eating habits, have not been established. Further studies are needed to determine the appropriate correction procedures for these measurements to be applied worldwide.

In conclusion, we reported that the skeletal muscle mass, the proportion of visceral fat to subcutaneous fat, and the age at the induction of anti-TNF therapy were associated with long-term clinical outcomes and that they may be helpful as prognostic markers in CD patients who have received anti-TNF therapy. Further investigations are needed to develop appropriate measurements and correction procedures for body composition parameters to be applied in various regions and races, and to determine the mechanisms of interaction between therapeutic responses and the body composition status, particularly through the crosstalk with myokines, adipokines and cytokines, which may contribute to the identification of new biomarkers and molecular targets for CD.

## Data Availability Statement

The data underlying this article will be shared on reasonable request to the corresponding author.

## Ethics Statement

The studies involving human participants were reviewed and approved by the Ethics Committee of Asahikawa Medical University (Registry Number: 18235). Written informed consent for participation was not required for this study in accordance with the national legislation and the institutional requirements.

## Author Contributions

KA and MF provided major input into the conceptual development of the studies, analyzed the collected data, wrote the manuscript, and supervised all of the investigations. KA, KU, YS, YK, YM, HS, TK, TS, KT, NU, and SK managed and treated the enrolled patients and collected the data. KM, HT, TO, and MF helped design the studies, interpret the data, and prepare/review the manuscript. All of the authors read and approved the final manuscript.

## Conflict of Interest

KA received lecture fees from Nippon Kayaku Co. Ltd., Janssen Pharmaceutical K.K., Mitsubishi Tanabe Pharma Corporation, Takeda Pharmaceutical Co. Ltd., AbbVie GK., JIMRO Co. Ltd., EA Pharma Co. Ltd., AYUMI Pharmaceutical Corporation, Mochida Pharmaceutical Co. Ltd., and Aspen Japan K.K. MF received lecture fees from Nippon Kayaku Co, Ltd., Janssen Pharmaceutical K.K., Mitsubishi Tanabe Pharma Corporation, Takeda Pharmaceutical Co. Ltd., AbbVie GK., JIMRO Co. Ltd., AYUMI Pharmaceutical Corporation, Pfizer Inc., EA Pharma Co. Ltd., Mochida Pharmaceutical Co. Ltd., Kyorin Pharmaceutical Co. Ltd., ZERIA Pharmaceutical Co. Ltd., and Aspen Japan K.K. and research grant from MOCHIDA Pharmaceutical Co. Ltd., Nippon Kayaku Co. Ltd., Pfizer Inc., Takeda Pharmaceutical Co. Ltd., Kamui Pharma Inc., and holds stocks of Kamui Pharma Inc. The remaining authors declare that the research was conducted in the absence of any commercial or financial relationships that could be construed as a potential conflict of interest.

## Publisher's Note

All claims expressed in this article are solely those of the authors and do not necessarily represent those of their affiliated organizations, or those of the publisher, the editors and the reviewers. Any product that may be evaluated in this article, or claim that may be made by its manufacturer, is not guaranteed or endorsed by the publisher.
