# Supplementary material for: Correlation Among Body Composition Parameters and Long-Term Outcomes in Crohn's Disease After Anti-TNF Therapy
Source: Front Nutr. 2022 Apr 1;9:765209. doi: 10.3389/fnut.2022.765209 (PMC9010511; doi:10.3389/fnut.2022.765209)
Supplement: Supplementary file 1 [file Data_Sheet_1.docx]

Supplementary Material

**
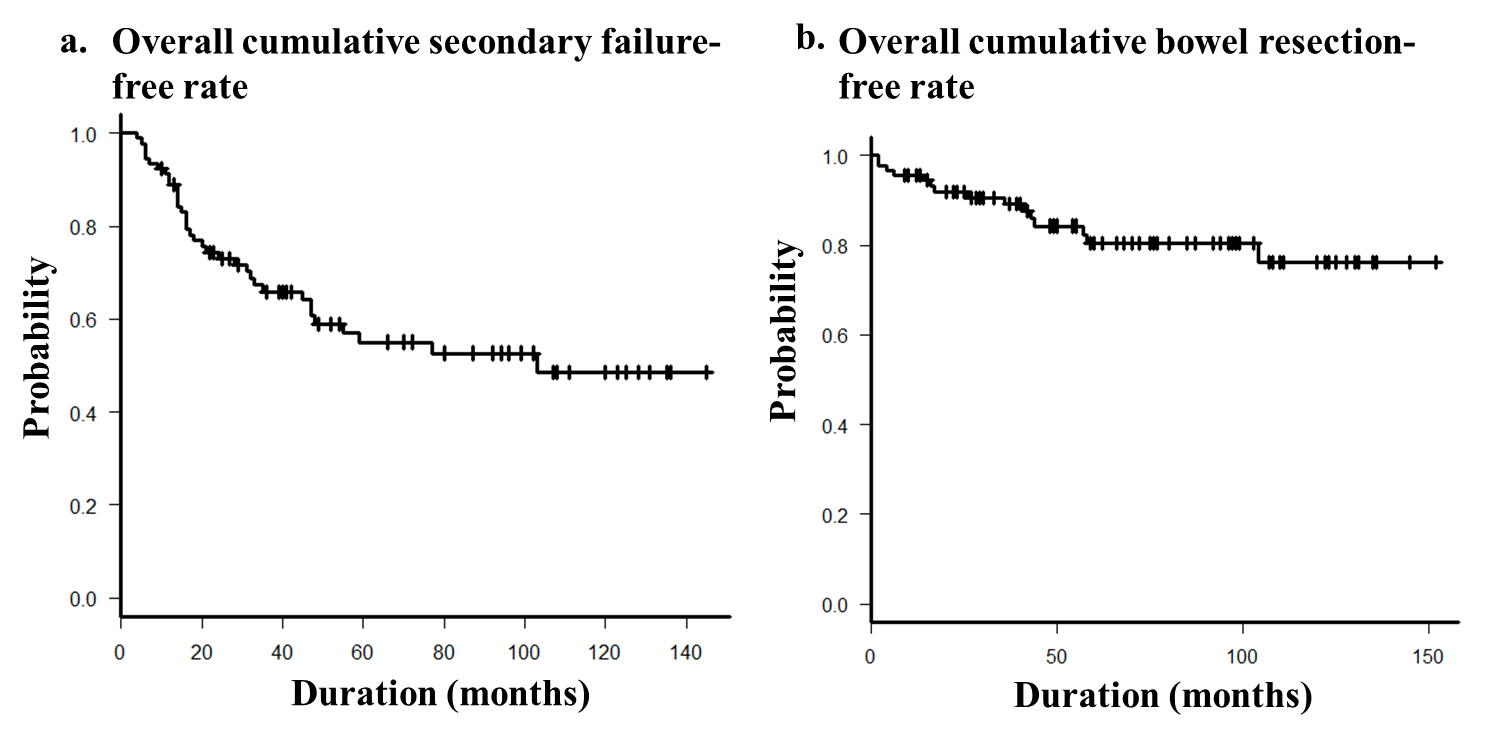
**

**Figure S1. Overall cumulative secondary failure-free (a) and bowel resection- free (b) rate.**

**
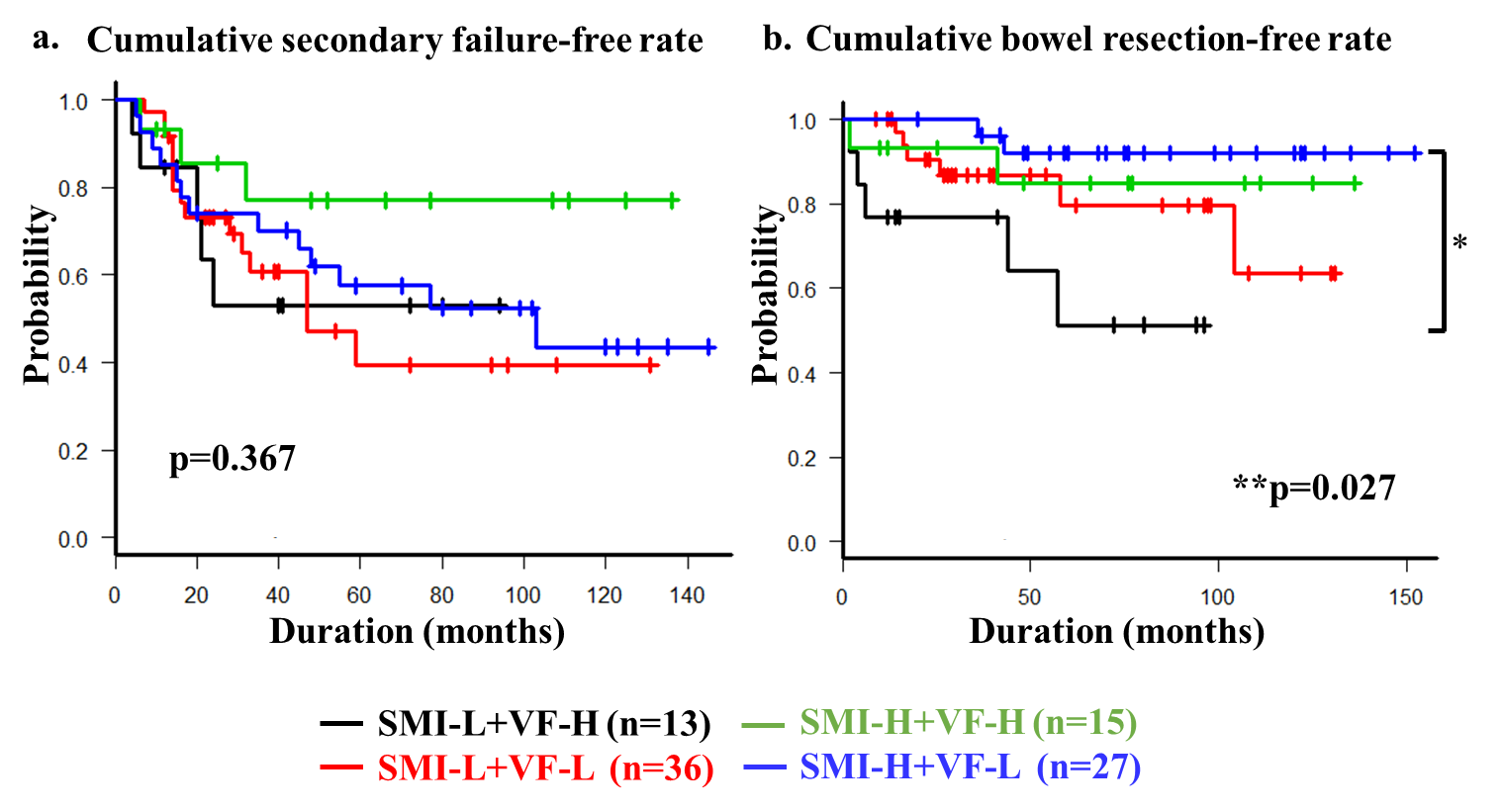
**

**Figure S2. Cumulative secondary failure-free (a) and bowel resection-free (b) rates classified according to the combination of the SMI and VF.**

**Table S1. Fat and muscle mass of the study population**

|  | Measurements |
| --- | --- |
| VFA [cm^2^] | 41.6 ± 38.1 (M; 44.6 ± 38.2, F; 32.1± 37.4) |
| SFA [cm^2^] | 65.0 ± 55.8 (M; 59.8 ± 54.9, F; 81.0± 56.4) |
| MFI | 0.82 ± 0.59 (M; 0.91 ± 0.54, F; 0.53± 0.64) |
| SMA [cm^2^] | 111.3 ± 30.5 (M; 122.1 ± 25.8, F; 77.6± 16.1) |
| SMI [cm^2^/m^2^] | 39.8 ± 9.1 (M; 42.6 ± 8.3, F; 31.1 ± 5.2) |
| PMA [cm^2^] | 15.7 ± 5.6 (M; 17.8 ± 4.4, F; 9.0± 2.8) |
| PMI [cm^2^/m^2^] | 5.5 ± 1.8 (M; 6.1 ± 1.6, F; 3.6 ± 1.0) |
| SMA/SFA | 3.5 ± 3.7 (M; 4.1 ± 4.0, F; 1.5 ± 1.0) |

VFA: Visceral fat area, SFA: Subcutaneous fat area, MFI: Mesenteric fat index, SMA: Skeletal muscle area, SMI: Skeletal muscle index, PMA: Psoas muscle area, PMI: Psoas muscle mass area
